# A scoping review of cognitive load assessment tools suitable for clinicians performing REBOA

**DOI:** 10.1186/s13049-025-01408-0

**Published:** 2025-07-09

**Authors:** Codey Simmons, Robbie Lendrum, Zane Perkins, Gareth Grier, Max Marsden

**Affiliations:** 1 Institute of Pre-Hospital Care, London’s Air Ambulance, London, UK; 2 Queen Mary University of London, London, UK; 3 London’s Air Ambulance, Barts Health NHS Trust, London, UK; 4 Barts Health NHS Trust, St Bartholomew’s Hospital, London, UK; 5 Centre for Trauma Sciences, Blizard Institute, Queen Mary University of London, London, UK; 6 Academic Department of Military Surgery and Trauma, Defence Medical Services, RCI, London, UK

**Keywords:** Cognitive load, Prehospital, REBOA

## Abstract

**Background:**

The ability to measure a clinician’s cognitive load allows task adaptions to optimise performance. The aim of this study was to identify cognitive load tools suitable for use by pre-hospital clinicians performing REBOA and develop a bespoke pre-hospital REBOA cognitive load assessment tool.

**Methods:**

A scoping review was conducted, following the PRISMA guidelines, to identify different cognitive load assessment tools in the literature from inception to January 2023. A qualitative narrative synthesis was used to compare tools based on their type, frequency of use, and context. Tools were assessed for contextual relevance and practical application to REBOA using defined criteria (CMTA-R score), created using domain experts.

**Results:**

Forty-nine articles were included for review, identifying 21 unique cognitive load tools: 10 subjective and 11 objective. The NASA-TLX was the most frequently used subjective tool (17 studies), scoring highest for potential REBOA use (CMTA-R 17). Heart Rate Variability (HRV) was the most common objective measure (14 studies), with a CMTA-R score of 13. A bespoke REBOA modification of the NASA-TLX, was suggested to quantify post-procedure cognitive load.

**Conclusions:**

This scoping review identifies the NASA-TLX and HRV as potential tools for assessing cognitive load during prehospital REBOA. A bespoke REBOA-adapted NASA-TLX, could be used post-procedure, while intra-procedural HRV monitoring could provide real-time data. Future research should validate this approach in clinical settings.

**Supplementary Information:**

The online version contains supplementary material available at 10.1186/s13049-025-01408-0.

## Introduction

The human brain is continually exposed to a myriad of sensory stimuli, processed for immediate action or conveyed into long-term memory. While long-term memory may be infinite, working memory is generally considered to have limited capacity [1, 2]. Cognitive load, sometimes referred to as bandwidth, describes the mental strain and effort required as working memory accumulates during a task. Cognitive overload may occur when the demands of a task outweigh an individual’s cognitive capacity [3]. Cognitive overload increases the risk of psychophysiological stress and medical error [4, 5]. Cognitive Load Theory divides cognitive capacity into three aspects: *intrinsic load,* related to the demands of the task itself; *extrinsic load*, related to the demands of the task environment; and *germane load*, the capacity required to construct long-term memory representations [6].

Resuscitative Endovascular Balloon Occlusion of the Aorta (REBOA) is used as a temporising measure to prevent exsanguination from non-compressible haemorrhage. For pre-hospital clinicians performing REBOA, the complex nature of the task, the challenging working environments and the high stakes decision making with limited information, generates significant cognitive load [7, 8]. Current REBOA research has predominantly focused on clinical application and survival rates. However, developing a deeper understanding of the cognitive demands of deploying REBOA could highlight areas that are mentally challenging for clinicians. Subsequently approaches to mitigate such challenges could then be developed.

Diverse strategies have been designed across many industries to quantify the amount of cognitive load a specific task requires. In medicine, measuring a clinician’s cognitive load enables tasks to be adapted to reduce intrinsic load, enhance environments to reduce extrinsic load and adapt training programmes to optimise germane load [3, 9]. To quantify a REBOA operator’s cognitive load, an appropriate tool must withstand both the variable contexts for REBOA and the dynamic course of a patient undergoing REBOA. This includes the extrinsic input from the varying pre-hospital environments alongside the intrinsic input of the procedure for differing patients with unique clinical pictures. This study aimed to conduct a scoping review of existing cognitive load tools to determine which tools would be suitable for pre-hospital clinicians performing REBOA. The findings will guide development of a pre-hospital REBOA cognitive load assessment tool.

## Methods

A scoping review of the published literature was conducted to identify cognitive load assessment strategies used in multistep medical procedures. Subjective and objective cognitive load assessment tools were sought. The study followed the PRISMA guidelines [10, 11].

### Search strategy

A literature search was undertaken for articles describing methods used to measure cognitive load of clinicians performing medical procedures. The following search terms were used: “methods”, “measuring”, “clinicians”, “cognitive load”, “haemorrhage control”, including variants for each field. PubMed, Embase, Scopus, Web of Science, Cochrane Library and IEEExplore databases were searched from inception to January 2023. The full search strategy can be viewed in Additional File 1 [see Additional File 1]. Peer-reviewed articles published in English were reviewed that described one or more cognitive load measurement tools used in simulated or real-world multistep procedures.

A procedure was defined as a multistep intervention requiring simultaneous decision-making and were performed prehospitally, in the Emergency Department, or in operating theatres. Systematic reviews and studies involving single-step or isolated decision-making tasks were excluded. Identified articles were screened for relevance by title then abstract. Those that remained underwent full manuscript review. Post review by a second reviewer, the literature that remained was read in-full for a second time. Data was extracted that included the study setting, the cognitive load measuring tool used, and the procedure performed.

### Data analysis

A qualitative narrative synthesis was conducted to compare the different tools identified to measure cognitive load. Data extracted from the included studies incorporated the type of tool, frequency and context in which it was used. To assess the methodological quality of the identified studies, the Medical Education Research Study Quality Instrument (MERSQI) was used [12]. This is a validated six-domain assessment tool covering 10 items, generating a score between three and 18, with higher scores denoting more rigorous research methods.

### Cognitive load measuring tool assessment for REBOA (CMTA-R)

The authors developed bespoke criteria to assess the contextual relevance and practical application of each tool. Operational factors impacting REBOA deployment were considered based on literature and the experiences of pre-hospital clinicians at London’s Air Ambulance [7, 13]. The **C**ognitive Load **M**easuring **T**ool **A**ssessment for **R**EBOA (CMTA-R) was used to score the applicability of subjective and objective cognitive load tools. The CMTA-R criteria can be found in Table 1. Assessment of subjective tools used criteria 1–14, whilst criteria 1,2,15–20 were applied to objective tools.

**Table 1:** Cognitive Load Measuring Tool Assessment for REBOA (CMTA-R) criteria for the assessment of subjective and objective tools

**Criteria**			**Score**	**Explanation**
**General Criteria**				
1	**Simulated/Real-world**	Simulated	1	Has the tool shown application in simulation and/or real-world scenarios?
		Real-world	2	
2	**Environment**	Theatre	1	What level of controlled environment was the tool used in?
		Emergency Department	2	
		Prehospital	3	
**Subjective Tool-specific Criteria**				
3	**Domains**	One	0	How many different cognitive load contributors were captured by the tool?
		Two	1	
		More than two	2	
4	**Rating scale**	Broad	1	Were rating scales broad (fewer increments) or provided more accurate representation (more increments)?
		Focused	2	
5	**Teamworking**	Yes	1	Did at least one tool domain encompass: teamworking?
		No	0	
6	**Decision making**	Yes	1	Did at least one tool domain encompass: decision making?
		No	0	
7	**Physical Exertion**	Yes	1	Did at least one tool domain encompass: physical exertion?
		No	0	
8	**Multitasking**	Yes	1	Did at least one tool domain encompass: multitasking?
		No	0	
9	**Situational awareness**	Yes	1	Did at least one tool domain encompass: situational awareness?
		No	0	
10	**Frustration**	Yes	1	Did at least one tool domain encompass: clinician’s frustration?
		No	0	
11	**Distraction**	Yes	1	Did at least one tool domain encompass: distractions from the task?
		No	0	
12	**Time pressure**	Yes	1	Did at least one tool domain encompass: time pressure of the task?
		No	0	
13	**Speed**	Yes	1	Did at least one tool domain encompass: the speed the task was completed?
		No	0	
14	**Procedural result**	Yes	1	Did at least one tool domain encompass: the final result of the procedure?
		No	0	
**Objective Tool-specific Criteria**				
15	**Wireless capability**	Yes	1	Does the tool demonstrate wireless capability?
		No	0	
16	**Restriction of equipment**	Non-restrictive	1	Is the equipment required for monitoring restrictive to the normal delivery of the task?
		Restrictive	0	
17	**Distraction from primary task**	Not distracting	1	Does the method of cognitive load measure provide added distraction from the task?
		Distracting	0	
18	**Learning curve**	Not required	1	Does the method of cognitive load measure require prior training before use?
		Required	0	
19	**Exclusive measurement**	Yes	1	Are measurements exclusive to the participant and not affected by environmental factors?
		No	0	
20	**Technological availability**	Commercial	2	Is the technology for measurement commercially available or bespoke for research purposes?
		Bespoke	1	

## Results

**Fig. 1:**
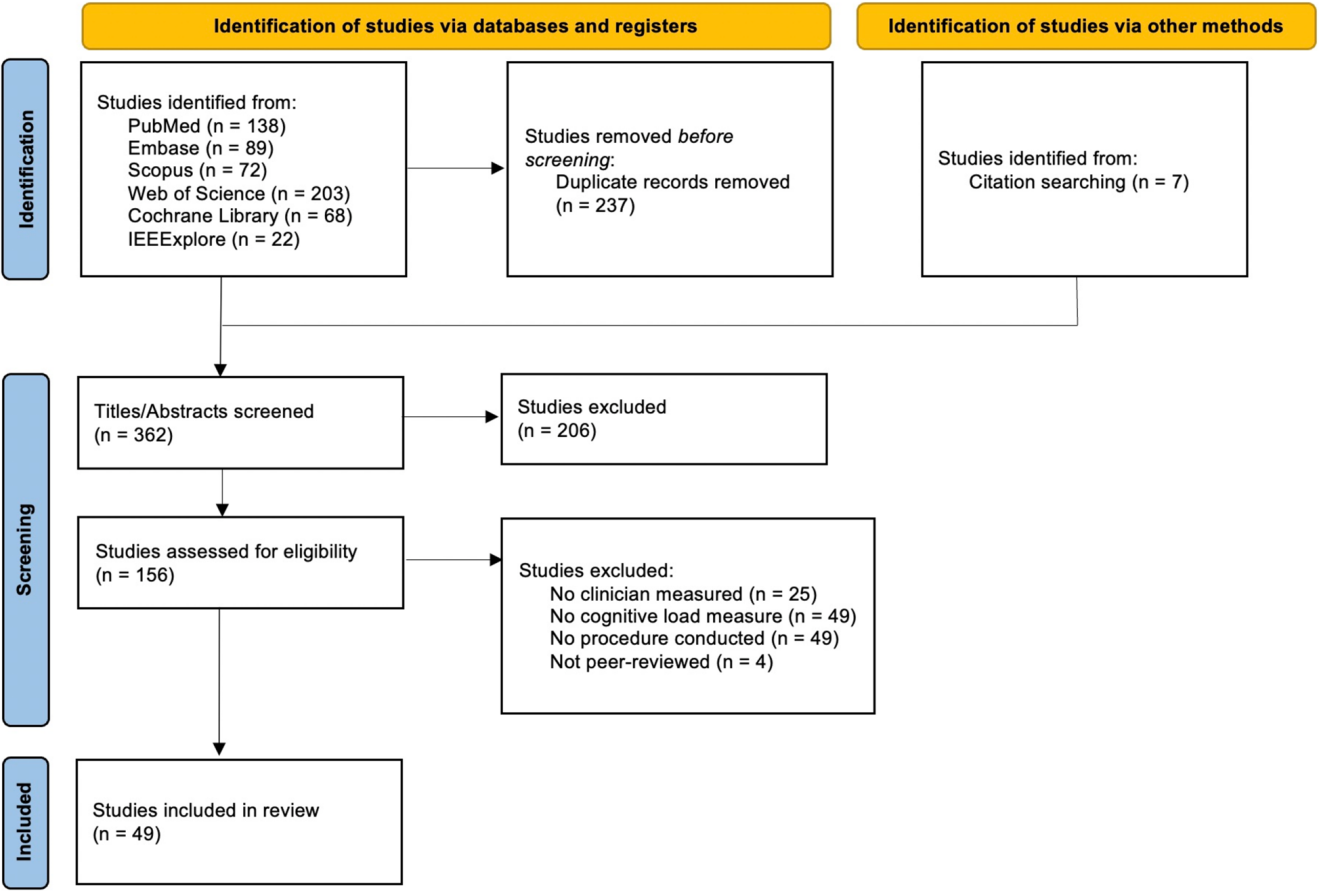
PRISMA flow diagram

The search identified 592 articles; an additional seven articles were identified from reference lists. From these, 362 were considered for screening, of which 49 met the inclusion criteria after full-text review. (Fig. 1) There were 21 different cognitive load assessment tools identified.

### Study design

Of the 49 included articles, 32 (65%) described a single cognitive load measurement tool while 17 (35%) used two or more. Subjective measures were used in 30 (61%) articles, objective measures in 29 (59%), and both subjective and objective approaches were used in 10 (20%) articles [14–23]. Most articles, 27 (55%), measured cognitive load in real world scenarios. Surgical procedures were the most common focus (40 articles), with nine studies utilising non-surgical scenarios. No articles included procedures performed pre-hospital.

### Subjective cognitive load tools

The review identified 10 distinct subjective cognitive load measuring tools. (Table 2) The National Aeronautics and Space Administration Task Load Index (NASA-TLX) was the most used subjective tool (17 articles) with an approximately even distribution between simulated (8/17) and real-world scenarios (9/17).

**Table 2:** Cognitive load measuring tools captured from the included literature. A description of each tool is included, along with the number of studies and study environments they were applied in and their CMTA-R score

**Type of Measure**		**Specific Tool**	**Description of Tool**	**Number of studies**			**CMTA-R**
				**Overall**	**Simulation**	**Real-world**	
Subjective		NASA-TLX	6 domains scored from 0–100 and weighted 0–5 including: mental demand, physical demand, temporal demand, performance, effort, frustration	17	8	4	17
		SURG-TLX	6 domains scored from 0–100 and weighted 0–5 including: mental demand, physical demand, temporal demand, task complexity, situational stress, distractions	8	1	7	15
		Likert Scales	5–7-point single domain scale ranging from “not stressful” to “extremely stressful”	3	1	2	5
		SMEQ	Single domain scored from 0–150 with 9 markers ranging from “not hard” to “extremely hard to do”	3	1	2	7
		HFEQ-CASS	38 questions scored on a 5-point scale: Block 1 = 12 questions: mental workload, surgical results, situational awareness, speed, risk taking. Block 2 = 26 questions: surgical and ergonomic characteristic	1	0	1	12
		MRQ	17 questions on 4-point scales assessing processing demand of: auditory, facial, manual, memory, spatial, tactile, visual and vocal	1	0	1	12
		SWAT	3 domains scored from ‘low, moderate and high’, measuring time load, mental effort and emotional stress	1	0	1	11
		Leppink Questionnaire	10 item questionnaire covering 3 domains of cognitive load: intrinsic load, extrinsic load, germane load	1	1	0	9
		STAI	40 questions on 4-point scales across 2 categories: ‘state’ and ‘trait’, focus on mental state and personal outlook	1	0	1	9
		OTAS	6-point scale across 5 domains: communication, coordination, cooperation, leadership, monitoring	1	0	1	8
Objective	Physiological Parameters	HRV	Statistical analysis of beat-to-beat variation on ECG	14	4	10	13
		Eye-tracking	Wearable monitor measuring pupillary dilation, tracking gaze duration and blink rate	5	3	2	11
		EEG	Electrodes on the scalp measure the voltage of electrical brain activity under different conditions determining functional area activation	3	3	0	9
		GSR	Sensory placed on the fingertips measuring skin conductance reflecting sweat production	3	3	0	8
		fNIR	Near-infrared spectroscopy for functional neuroimaging by estimating the haemodynamic activity of the brain	1	0	1	7
		EMG	Electrodes positioned on the hands or arms delivering a small impulse to measure nerve conduction	1	1	0	6
		Oxygen Saturations	Measure of peripheral SpO2% using a pulse oximeter	1	0	1	6
		Heat Flux	Thermal imaging of heat distribution within the face	1	1	0	5
		IOP	Use of rebound tonometry as a measure of IOP in both eyes pre- and post-procedure	1	1	0	5
	Procedural Parameters	Error Rate	Analysis of each procedural step, recording correct execution and number of errors	1	1	0	9
	Secondary Task Analysis	Reaction Time	Secondary stimulus (auditory/tactile) delivered at varying intervals during the primary procedure, reaction times measured to identify and terminate the stimulus	6	6	0	6

First published in 1988, the NASA-TLX is often presented as a post-hoc questionnaire providing a weighted score across six domains, three measuring the burden of the task itself: *Mental demand*, *Physical demand*, *Temporal demand*; and three measuring the task environment: *Performance*, *Effort* and *Frustration* [24]. The NASA-TLX scored the highest for potential transferability to prehospital REBOA (CMTA-R = 17) (Table 2). SURG-TLX, a surgery-specific variant of the NASA-TLX, was the second highest scoring tool for both frequency of use and CMTA-R (CMTA-R = 15). SURG-TLX was used in eight studies, one (13%) simulated and seven (88%) real-world. The SURG-TLX differs to the NASA-TLX by substituting *Performance*, *Effort* and *Frustration* domains for *Task Complexity* (“How complex was the procedure?”), *Situational Stress* (“How anxious did you feel whilst performing the procedure?”), and *Distractions* (“How distracting was the operating environment?”) [25].

Other than the NASA- and SURG-TLXs, only two other types of subjective measurement were cited in more than one article: the Subjective Mental Effort Questionnaire (SMEQ) and several tools employing adapted Likert Scales for specific uses [14, 15, 26–29]. Both SMEQ and Likert scales utilise a single domain approach.

### Objective tools

The review identified 11 objective measuring tools cited in 23 (62%) simulated and 14 (38%) real-world scenarios (Table 2). The most common objective tool was Heart Rate Variability (HRV) (14 articles). HRV assesses the changes in duration between adjacent heartbeats through electrocardiogram (ECG) monitoring. Divided into time and frequency domains, it provides a physiological representation of cognitive stress reflecting the neurocardiac axis and various cardiac inputs [30]. Monitoring may be achieved through 3-lead ECGs, commercial heart rate monitors and smart watches. [31, 32]. HRV was the highest scoring objective tool, showing the greatest potential for use with REBOA operators (CMTA-R = 13) (Table 2).

Another widely used physiological objective tool used eye-tracking (CMTA-R = 11). Used in five studies, eye-tracking represents autonomic responses through measure of pupil size, gaze duration and blink frequency [15, 16, 33]. Monitoring included both wearable and static devices.

Objective measurements of cognitive load can also be achieved through procedural and situational parameters. Alternative objective measures were used across seven articles. Secondary Task Analysis was the most frequently used tool reflecting this approach. In six studies, all under simulated conditions, Secondary Task Analysis tested the reaction speeds of a clinician in termination of an additional stimulus whilst undertaking a procedure [21, 34–39]. This aims to test task-focus. Secondary Task Analysis scored lower on the CMTA-R (CMTA-R = 6).

### Participants and study designs

In the included studies the number of participants varied from one to over 100. Surgeons were the most studied (39 studies), followed by anaesthetists (7 studies) and emergency medicine physicians (6 studies). A combined total of 13 randomised controlled trials were recorded. Real-world settings were used in six studies [25, 27, 29, 32, 40, 41], with seven randomised controlled trials occurring in simulation [37, 39, 42–46].

The MERSQI score used for bias assessment varied from 15 to nine, with an average score of 13. Overall, the highest scoring studies included randomised control trials (13 studies) and studies recording real-world patient outcomes (17 studies). MERSQI scores for each article can be found in the summary table of included literature [see Additional File 2].

## Discussion

The review has identified a multitude of subjective and objective cognitive load measuring tools with potential application to REBOA operators. Analysis of the benefits and limitations of each tool highlighted the NASA-TLX and HRV as the most suitable subjective and objective tools to assess cognitive load of REBOA operators. Using both tools in conjunction would likely support the idea that intra-procedure monitoring could validate post-procedure questionnaire results.

Pre-hospital REBOA is a time-critical, high-acuity, low-frequency procedure which by occluding the aorta increases cerebral and coronary perfusion. These effects are desirable in both traumatic exsanguination and non-traumatic cardiac arrest. The use of REBOA as a cardiopulmonary resuscitation (CPR) adjunct has been shown to increase rates of Return of Spontaneous Circulation (ROSC) [47, 48]. Meanwhile, when deployed for exsanguinating patients from sub-diaphragmatic haemorrhage, REBOA aids patient survival during transfer to definitive haemostasis [49, 50].

Achieving aortic occlusion with REBOA is a complex process with risk. Significant challenges include patient selection and the fundamental step of achieving femoral arterial access. Obtaining arterial access in the pre-hospital environment on patients either close to exsanguination or with ongoing chest compressions is technically extremely difficult. Potential challenges include visualising the artery under ultrasound and successful guide wire placement. The REBOA operator will be aware that failed attempts increase on-scene time and decrease the probability of patient survival [51].

Another technique that introduces complexity is the use of partial-REBOA (pREBOA). In trauma, pREBOA has the potential to increase total duration of safe aortic occlusion, reduce inflammatory complications and improve patient outcomes [49]. However, the technical application of partial aortic occlusion is challenging as the therapeutic window is narrow with current REBOA technology. The key issue relates to the amount of volume that can be removed from the occluding aortic balloon before an exponential increase in aortic flow distal to the balloon. The meticulously planned process of achieving pREBOA is complicated further by the phenomenon of physiological pREBOA. The dynamic nature of the aorta, affected by catecholamines and fluid status, causes vasoconstriction and dilation leading to partial occlusion regardless of the clinician’s intentions [52, 53]. Such unwanted increases in distal flow can cause proximal collapse of blood pressure, cardiac instability and further bleeding [53].

Technological advancements present opportunities to streamline the aspects of REBOA clinicians find challenging. AI-guided ultrasound and preloaded guide wires offer a potential to improve arterial access [51]. Meanwhile, automated pREBOA systems, like Endovascular Variable Aortic Control (EVAC), can independently adjust aortic balloon inflation based on real-time pressure changes, potentially improving patient outcomes [54, 55].

Such technological advancements could not only enhance procedural efficacy but also reduce the cognitive load on clinicians. By automating technically demanding tasks, pre-hospital clinicians can allocate more cognitive bandwidth to other critical aspects of patient care. While current research into REBOA has necessarily focused on feasibility, processes and survival rates, a deeper understanding of the cognitive demands of REBOA is crucial [52]. By quantifying a clinician’s cognitive load, we can assess the value of technological improvements and optimise skill-uptake during training. Regular simulation training has been shown to reduce cognitive burden, widening the clinician’s capacity for learning [56, 57]. Therefore, measuring cognitive load can guide both reductions in task complexity and the delivery of frequent high-quality training, ultimately improving practice and clinical decision-making in high-stress pre-hospital environments.

This study demonstrates a combination of both subjective and objective cognitive load measuring tools appears best suited to REBOA operators. Over 20% of the included articles used both types of measure, half of which demonstrated real-world application. The synergy between a real-time physiological or procedural marker and a retrospective post-hoc questionnaire, allows for correlations to be drawn between procedural steps and peaks in mental strain. This would be beneficial in the context of REBOA. By identifying the steps incurring the highest cognitive burden, these aspects may be the focus for procedural adaptations and further training.

The cognitive load assessment tools captured in the review were assessed using bespoke criteria for contextual relevance to pre-hospital REBOA (CMTA-R). The CMTA-R criteria was developed by the authors, using domain expertise and real-world experience of pre-hospital physicians deploying REBOA. The authors focused on a score that included operational factors impacting the delivery of the procedure. This approach provided a simple method to compare cognitive load tools using the same benchmarking approach where no such validated tool existed. However, it should be noted that the factors used to rate the cognitive load tools were all derived from pre-hospital clinicians working in one institution. As such, some factors may have been neglected and others over-emphasised.

The NASA-TLX, a versatile tool for assessing cognitive load, is well-suited for REBOA operators. Its six-domain approach, applicable to both real-world and simulated settings, provides a comprehensive understanding of cognitive burden. The weighted scales and adaptability of the NASA-TLX make it ideal for capturing the unique demands of REBOA in diverse operational environments. REBOA performed in different contexts may present differing levels of complexity. For example, using REBOA for trauma in cities has different challenges and mental demands than using it for cardiac arrest in rural areas. Factors such as the duration of balloon inflation, geographical distances travelled, and hospital selection are all affected by context. A useful cognitive load tool will capture and quantify this complexity in its ability to measure cognitive load. For example, by using the NASA-TLX, these differing complexities in decision making would be captured in the Mental Demand domain.

A bespoke REBOA cognitive load assessment questionnaire, building on the NASA-TLX framework, could further refine the tool for pre-hospital settings, focusing on unique task-based and environmental factors. An assessment tool of this sort could be widely accessed and implemented across pre-hospital services currently deploying REBOA. Use of a post-procedure cognitive load questionnaire, such as the NASA-TLX or a bespoke REBOA variant, could generate data helping services guide changes to improve performance for future REBOA patients [58].

This study highlights the adaptability of the NASA-TLX for assessing cognitive load across diverse clinical contexts. By tailoring intrinsic and extrinsic factors to specific procedures (e.g. task complexity in Resuscitative Thoracotomy or image interpretation in Point-of-Care Ultrasound), clinicians gain valuable insights into their cognitive load during challenging interventions like Extra Corporeal Membrane Oxygenation (ECMO) cannulation or pre-hospital REBOA. This enhanced awareness can inform strategies to mitigate cognitive overload and optimise patient care.

HRV, a validated physiological marker of cognitive load, is a promising objective tool for assessing REBOA operators. Previous studies of performance have shown heart rates above 140-145bpm can be associated with inhibition of working memory [8, 59]. Its non-invasive nature and compatibility with wearable devices make it suitable for pre-hospital settings. Unlike other methods, such as Functional Near-Infrared Spectroscopy (fNIR), Electroencephalography (EEG), or Electromyography (EMG), HRV does not require bulky equipment or restrictive setups, allowing for continuous monitoring during procedures.

HRV can be correlated with subjective tools like the NASA-TLX to provide a comprehensive understanding of cognitive burden [23, 60]. A recent case report demonstrated the feasibility of using a commercial smartwatch, to measure HRV, and the NASA-TLX to assess cognitive load in pre-hospital clinicians performing a Resuscitative Thoracotomy [61]. This case report highlights the potential for combining objective and subjective measures in real-world pre-hospital settings.

The present study proposes a novel cognitive load assessment tool for REBOA operators, combining a REBOA-specific NASA-TLX questionnaire and intra-procedural HRV monitoring. This approach offers the first attempt to quantify clinicians’ cognitive load for a pre-hospital procedure. A feasibility study would be required to validate this approach.

### Limitations

Cognitive load measuring tools have been utilised throughout many high-stakes professions. The present study only reviewed clinical usage. Tools from other occupational domains may also be suitable. Their assessment would require further research. Overlap in the use of key search terms, such as “*mental stress*”, “*mental effort*”, “*cognitive load*”, and “*bandwidth*”, may also lead to confounding of different concepts for which these terms are also used. The present study focuses on metrics associated with cognitive load compared to other cognitive states such as psychological trauma and emotional stress. Additionally, the captured literature did not represent pre-hospital REBOA directly. The use of CMTA-R provided a standardised comparative tool to prevent bias when assessing measures in context. However, this approach itself may be limited as factors used to rate the captured tools were derived from pre-hospital clinicians working in one institution.

## Conclusion

This scoping review identifies the NASA-TLX and HRV as potential tools for assessing cognitive load during prehospital REBOA. This dual assessment approach could be achieved through development of a bespoke REBOA-adapted NASA-TLX, used post-procedure, with intra-procedural HRV monitoring providing real-time data. Future research should validate this approach in clinical settings. This study provides the first step in obtaining cognitive load measures for prehospital REBOA operator.

## Supplementary Information

Supplementary Material 1

Supplementary Material 2: [62–78]

## Data Availability

No datasets were generated or analysed during the current study.

## Abbreviations

AVR: Aortic Valve Repair
CABG: Coronary Artery Bypass Graft
CMTA-R: Cognitive Load
CPR: Cardiopulmonary Resuscitation
ECG: Electrocardiogram
ECMO: Extra Corporeal Membrane Oxygenation
EEG: Electroencephalography
EM: Emergency Medicine
EMG: Electromyography
EVAC: Endovascular Variable Aortic Control
fNIRS: Functional Near-Infrared Spectroscopy
GSR: Galvanic Skin Response
HFEQ-CASS: Human Factors Evaluation Questionnaire for Computer Assisted Surgery Systems
HRV: Heart Rate Variability
IOP: Intraocular pressure
MERSQI: Medical Education Research Study Quality Instrument
MRQ: Multiple Resource Questionnaire
NASA-TLX: National Aeronautics and Space Administration Task Load Index
OTAS: Observational Teamwork Assessment for Surgery
REBOA: Resuscitative Endovascular Balloon Occlusion of the Aorta
ROSC: Return of Spontaneous Circulation
SMEQ: Subjective Mental Effort Questionnaire
SpO2%: Oxygen Saturations
STAI: State-Trait Anxiety Inventory
SURG-TLX: Surgical Task Load Index
SWAT: Subjective Workload Assessment Technique

## References

[1] Cowan N. Working Memory Underpins Cognitive Development, Learning, and Education. Educ Psychol Rev. 2014Jun 1;26(2):197–223. 25346585 10.1007/s10648-013-9246-y PMC4207727

[2] Sweller J. Cognitive load during problem solving: Effects on learning. Cogn Sci. 1988Apr 1;12(2):257–85.

[3] Sweller J. Cognitive load theory, learning difficulty, and instructional design. Learn Instr. 1994Jan 1;4(4):295–312.

[4] Dias RD, Ngo-Howard MC, Boskovski MT, Zenati MA, Yule SJ. Systematic review of measurement tools to assess surgeons’ intraoperative cognitive workload. Br J Surg. 2018Apr;105(5):491–501. 29465749 10.1002/bjs.10795 PMC5878696

[5] Vella KM, Hall AK, van Merrienboer JJG, Hopman WM, Szulewski A. An exploratory investigation of the measurement of cognitive load on shift: Application of cognitive load theory in emergency medicine. AEM Educ Train. 2021Aug;5(4): e10634. 34447896 10.1002/aet2.10634 PMC8372981

[6] Sweller J, van Merrienboer JJG, Paas FGWC. Cognitive Architecture and Instructional Design. Educ Psychol Rev. 1998Sep 1;10(3):251–96.

[7] Price R, Bendall JC, Patterson JA, Middleton PM. What causes adverse events in prehospital care? A human-factors approach Emerg Med J. 2013Jul;30(7):583–8. 22802456 10.1136/emermed-2011-200971

[8] Maleczek M, Schebesta K, Hamp T, Burger AL, Pezawas T, Krammel M, et al. ST-T segment changes in prehospital emergency physicians in the field: a prospective observational trial. Scand J Trauma Resusc Emerg Med. 2022Jul;15(30):47. 10.1186/s13049-022-01033-1 PMC9288087 35841049

[9] Fraser KL, Ayres P, Sweller J. Cognitive Load Theory for the Design of Medical Simulations. Simul Healthc. 2015Oct;10(5):295. 26154251 10.1097/SIH.0000000000000097

[10] Page MJ, McKenzie JE, Bossuyt PM, Boutron I, Hoffmann TC, Mulrow CD, et al. The PRISMA 2020 statement: an updated guideline for reporting systematic reviews. BMJ. 2021Mar;29(372): n71. 10.1136/bmj.n71 PMC8005924 33782057

[11] Page MJ, Moher D, Bossuyt PM, Boutron I, Hoffmann TC, Mulrow CD, et al. PRISMA 2020 explanation and elaboration: updated guidance and exemplars for reporting systematic reviews. BMJ. 2021Mar;29(372): n160. 10.1136/bmj.n160 PMC8005925 33781993

[12] Association Between Funding and Quality of Published Medical Education Research | Medical Journals and Publishing | JAMA | JAMA Network [Internet]. [cited 2023 Apr 11]. Available from: https://jamanetwork.com/journals/jama/fullarticle/208628

[13] Brenner ML, Moore LJ, DuBose JJ, Tyson GH, McNutt MK, Albarado RP, et al. A clinical series of resuscitative endovascular balloon occlusion of the aorta for hemorrhage control and resuscitation. J Trauma Acute Care Surg. 2013Sep;75(3):506. 24089121 10.1097/TA.0b013e31829e5416

[14] Pimentel G, Rodrigues S, Silva PA, Vilarinho A, Vaz R, Silva Cunha JP. A wearable approach for intraoperative physiological stress monitoring of multiple cooperative surgeons. Int J Med Inf. 2019Sep;129:60–8. 10.1016/j.ijmedinf.2019.05.028 31445290

[15] Kapp C, Akulian J, Yu D, Chen A, Cardenas-Garcia J, Molena D, et al. Cognitive Load in Electromagnetic Navigational and Robotic Bronchoscopy for Pulmonary Nodules. Sch. 2021Mar;2(1):97–107. 10.34197/ats-scholar.2020-0033OC PMC8043265 33870326

[16] Anschuetz L, Niederhauser L, Wimmer W, Yacoub A, Weibel D, Mast FW, et al. Comparison of 3- vs 2-Dimensional Endoscopy Using Eye Tracking and Assessment of Cognitive Load Among Surgeons Performing Endoscopic Ear Surgery. JAMA Otolaryngol-- Head Neck Surg. 2019;145(9):838–45. 31343675 10.1001/jamaoto.2019.1765 PMC6659156

[17] Kelkar A, Kelkar J, Chougule Y, Bolisetty M, Singhvi P. Cognitive workload, complications and visual outcomes of phacoemulsification cataract surgery: Three-dimensional versus conventional microscope. Eur J Ophthalmol. 2022Sep;32(5):2935–41. 34825825 10.1177/11206721211062034

[18] Pappada S, Papadimos T, Lipps J, Feeney J, Durkee K, Galster S, et al. Establishing an instrumented training environment for simulation-based training of health care providers: An initial proof of concept. Int J Acad Med. 2016Jan;2(1):32–40.

[19] Merkle F, Kurtovic D, Starck C, Pawelke C, Gierig S, Falk V. Evaluation of attention, perception, and stress levels of clinical cardiovascular perfusionists during cardiac operations: a pilot study. Perfus-UK. 2019Oct;34(7):544–51. 10.1177/0267659119828563 30868941

[20] Dias RD, Conboy HM, Gabany JM, Clarke LA, Osterweil LJ, Arney D, et al. Intelligent Interruption Management System to Enhance Safety and Performance in Complex Surgical and Robotic Procedures. 20 Context-Aware Oper Theaters Comput Assist Robot Endosc Clin Image-Based Proced Skin Image Anal First Int Workshop 20 2018 5th Int Workshop CARE 2018 7th Int. 2018 Sep;11041:62–8. 10.1007/978-3-030-01201-4_8 PMC6267949 30506066

[21] Britt RC, Scerbo MW, Montano M, Kennedy RA, Prytz E, Stefanidis D. Intracorporeal suturing: Transfer from Fundamentals of Laparoscopic Surgery to cadavers results in substantial increase in mental workload. Surgery. 2015Nov;158(5):1428–33. 26003907 10.1016/j.surg.2015.03.032

[22] Vera J, Diaz-Piedra C, Jiménez R, Sanchez-Carrion JM, Di Stasi LL. Intraocular pressure increases after complex simulated surgical procedures in residents: an experimental study. Surg Endosc. 2019Jan;33(1):216–24. 29967993 10.1007/s00464-018-6297-7

[23] Joseph B, Parvaneh S, Swartz T, Haider AA, Hassan A, Kulvatunyou N, et al. Stress among surgical attending physicians and trainees: A quantitative assessment during trauma activation and emergency surgeries. J Trauma Acute Care Surg. 2016Oct;81(4):723–8. 27389128 10.1097/TA.0000000000001162

[24] Huckaby LV, Cyr AR, Handzel RM, Littleton EB, Crist LR, Luketich JD, et al. Postprocedural Cognitive Load Measurement With Immediate Feedback to Guide Curriculum Development. Ann Thorac Surg. 2022Apr;113(4):1370–7. 34214548 10.1016/j.athoracsur.2021.05.086 PMC8991377

[25] Bingener J, Mohamed AO, Lowndes BR, McConico AL, Hallbeck S. Modified NASA workload tool identifies physical and cognitive surgeon workload for laparoscopic procedures. J Am Coll Surg. 2014;219(4): e13.

[26] López-Cano M, Pereira JA, Mojal S, Lozoya R, Quiles MT, Arbós MA, et al. An ergonomic study of single-port versus multi-port laparoscopic mesh insertion for ventral hernia repair. Eur Surg Res Eur Chir Forsch Rech Chir Eur. 2012;49(3–4):107–12. 10.1159/000342925 23095250

[27] Kumar P, Mishra TS, Sarthak S, Sasmal PK. Lithotomy versus prone position for perianal surgery: a randomized controlled trial. Ann Coloproctology. 2022Apr;38(2):117–23. 10.3393/ac.2020.12.16 PMC9021856 34098632

[28] Weinger MB, Vredenburgh AG, Schumann CM, Macario A, Williams KJ, Kalsher MJ, et al. Quantitative description of the workload associated with airway management procedures. J Clin Anesth. 2000Jun;12(4):273–82. 10960198 10.1016/s0952-8180(00)00152-5

[29] Grochola LF, Soll C, Zehnder A, Wyss R, Herzog P, Breitenstein S. Robot-assisted single-site compared with laparoscopic single-incision cholecystectomy for benign gallbladder disease: results of a singleblinded randomized controlled trial. HPB. 2018;1(20):S726. 10.1186/s12893-017-0206-1 PMC5301379 28183345

[30] Shaffer F, Ginsberg JP. An Overview of Heart Rate Variability Metrics and Norms. Front Public Health. 2017Sep;28(5):258. 10.3389/fpubh.2017.00258 PMC5624990 29034226

[31] Park SH, Goldberg SA, Al-Ballaa A, Tayeb B, Basurrah M, Abahuje E, et al. Objective Measurement of Learners’ Cognitive Load During Simulation-Based Trauma Team Training: A Pilot Study. J Surg Res. 2022Nov;279:361–7. 35816846 10.1016/j.jss.2022.06.023

[32] Böhm B, Rötting N, Schwenk W, Grebe S, Mansmann U. A prospective randomized trial on heart rate variability of the surgical team during laparoscopic and conventional sigmoid resection. Arch Surg Chic Ill 1960. 2001 Mar;136(3):305–10. 10.1001/archsurg.136.3.305 11231850

[33] Shafiei SB, Hussein AA, Ahmed Y, Guru K. Can eye tracking help explain an expert surgeon’s brain performance during robot-assisted surgery? J Urol. 2018;199(4):e1-2.

[34] Yang J, Barragan JA, Farrow JM, Sundaram CP, Wachs JP, Yu D. An Adaptive Human-Robotic Interaction Architecture for Augmenting Surgery Performance Using Real-Time Workload Sensing-Demonstration of a Semi-autonomous Suction Tool. Hum Factors. 2022Nov;11:187208221129940. 10.1177/00187208221129940 PMC11558698 36367971

[35] Lowe DJ, James SA, Lloyd A, Clegg GR. Feasibility of EEG to monitor cognitive performance during venous cannulation: EEG Distracted Intravenous Access (E-DIVA). BMJ Simul Technol Enhanc Learn. 2016;2(3):68–72. 35519423 10.1136/bmjstel-2015-000082 PMC8936945

[36] Kennedy-Metz L, Dias R, Srey R, Rance G, Furlanello C, Zenati M. Sensors for Continuous Monitoring of Surgeon’s Cognitive Workload in the Cardiac Operating Room. Sensors. 2020;20(22):6616. 10.3390/s20226616. 33227967 10.3390/s20226616 PMC7699221

[37] Frederiksen J, Sorensen S, Konge L, Svendsen M, Nobel-Jorgensen M, Bjerrum F, et al. Cognitive load and performance in immersive virtual reality versus conventional virtual reality simulation training of laparoscopic surgery: a randomized trial. Surg Endosc Interv Tech. 2020Mar;34(3):1244–52. 10.1007/s00464-019-06887-8 31172325

[38] Andersen SAW, Mikkelsen PT, Konge L, Cayé-Thomasen P, Sørensen MS. Cognitive Load in Mastoidectomy Skills Training: Virtual Reality Simulation and Traditional Dissection Compared. J Surg Educ. 2016;73(1):45–50. 26481267 10.1016/j.jsurg.2015.09.010

[39] Boet S, Sharma B, Pigford A, Hladkowicz E, Rittenhouse N, Grantcharov T. Debriefing decreases mental workload in surgical crisis: A randomized controlled trial. SURGERY. 2017May;161(5):1215–20. 28104293 10.1016/j.surg.2016.11.031

[40] Stelter K, Ertl-Wagner B, Luz M, Muller S, Ledderose G, Siedek V, et al. Evaluation of an image-guided navigation system in the training of functional endoscopic sinus surgeons. A prospective, randomised clinical study. Rhinology. 2011;49(4):429–37. 21991568 10.4193/Rhino11.035

[41] Heemskerk J, Zandbergen HR, Keet SWM, Martijnse I, van Montfort G, Peters RJA, et al. Relax, it’s just laparoscopy! A prospective randomized trial on heart rate variability of the surgeon in robot-assisted versus conventional laparoscopic cholecystectomy. Dig Surg. 2014;31(3):225–32. 25277215 10.1159/000365580

[42] Chowriappa A, Raza SJ, Fazili A, Field E, Malito C, Samarasekera D, et al. Augmented-reality-based skills training for robot-assisted urethrovesical anastomosis: a multi-institutional randomised controlled trial. BJU Int. 2015Feb;115(2):336–45. 24612471 10.1111/bju.12704

[43] Dixon BJ, Daly MJ, Chan H, Vescan A, Witterick IJ, Irish JC. Augmented real-time navigation with critical structure proximity alerts for endoscopic skull base surgery. Laryngoscope. 2014Apr;124(4):853–9. 24122916 10.1002/lary.24385

[44] Do preparatory online modules optimize cognitive load during simulated resuscitation scenarios? | Cochrane Library [Internet]. [cited 2023 Jan 21]. Available from: https://www.cochranelibrary.com/central/doi/10.1002/central/CN-02274008/full

[45] Pluyter JR, Rutkowski AF, Jakimowicz JJ. Immersive training: breaking the bubble and measuring the heat. Surg Endosc. 2014May;28(5):1545–54. 24399519 10.1007/s00464-013-3350-4

[46] Fenik Y, Celebi N, Wagner R, Nikendei C, Lund F, Zipfel S, et al. Prepackaged central line kits reduce procedural mistakes during central line insertion: a randomized controlled prospective trial. BMC Med Educ. 2013Apr;30(13):60. 10.1186/1472-6920-13-60 PMC3645964 23631396

[47] A protocol for the ERICA-ARREST feasibility study of Emergency Resuscitative Endovascular Balloon occlusion of the Aorta in Out-of-Hospital Cardiac Arrest - ScienceDirect [Internet]. [cited 2025 Apr 7]. Available from: https://www.sciencedirect.com/science/article/pii/S2666520424001395 10.1016/j.resplu.2024.100688 PMC11225899 38974930

[48] Gedeborg R, Rubertsson S, Wiklund L. Improved haemodynamics and restoration of spontaneous circulation with constant aortic occlusion during experimental cardiopulmonary resuscitation. Resuscitation. 1999May 1;40(3):171–80. 10395400 10.1016/s0300-9572(99)00021-0

[49] Marsden M, Lendrum R, Davenport R. Revisiting the promise, practice and progress of resuscitative endovascular balloon occlusion of the aorta. Curr Opin Crit Care. 2023Dec 1;29(6):689–95. 37861182 10.1097/MCC.0000000000001106

[50] Lendrum R, Perkins Z, Chana M, Marsden M, Davenport R, Grier G, et al. Pre-hospital Resuscitative Endovascular Balloon Occlusion of the Aorta (REBOA) for exsanguinating pelvic haemorrhage. Resuscitation. 2019Feb;1(135):6–13. 10.1016/j.resuscitation.2018.12.018 30594600

[51] Marsden M, Lendrum R, Perkins Z, Davenport RA. REBOA for remote damage control resuscitation and the race against time. Curr Opin Anesthesiol. 2025Apr;38(2):100. 10.1097/ACO.0000000000001474 39937037

[52] Lendrum RA, Perkins Z, Marsden M, Cochran C, Davenport R, Chege F, et al. Prehospital Partial Resuscitative Endovascular Balloon Occlusion of the Aorta for Exsanguinating Subdiaphragmatic Hemorrhage. JAMA Surg. 2024Sep 1;159(9):998–1007. 38985496 10.1001/jamasurg.2024.2254 PMC11238066

[53] Johnson MA, Neff LP, Williams TK, DuBose JJ. Partial Resuscitative Balloon Occlusion of the AORTA (PREBOA): Clinical Technique and Rationale. J Trauma Acute Care Surg. 2016;81(5 Suppl 2 Proceedings of the 2015 Military Health System Research Symposium):S133–137. 10.1097/TA.0000000000001146. 10.1097/TA.0000000000001146 PMC8789541 27244578

[54] Williams TK, Neff LP, Johnson MA, Russo RM, Ferencz SA, Davidson AJ, et al. Extending REBOA: Endovascular Variable Aortic Control (EVAC) in a Lethal Model of Hemorrhagic Shock. J Trauma Acute Care Surg. 2016Aug;81(2):294–301. 27070441 10.1097/TA.0000000000001075 PMC4961606

[55] Williams TK, Tibbits EM, Hoareau GL, Simon MA, Davidson AJ, DeSoucy ES, et al. Endovascular variable aortic control (EVAC) versus resuscitative endovascular balloon occlusion of the aorta (REBOA) in a swine model of hemorrhage and ischemia reperfusion injury. J Trauma Acute Care Surg. 2018Sep;85(3):519. 30142105 10.1097/TA.0000000000002008

[56] Haji FA, Khan R, Regehr G, Drake J, de Ribaupierre S, Dubrowski A. Measuring cognitive load during simulation-based psychomotor skills training: sensitivity of secondary-task performance and subjective ratings. Adv Health Sci Educ Theory Pract. 2015Dec;20(5):1237–53. 25761454 10.1007/s10459-015-9599-8

[57] Maimon NB, Bez M, Drobot D, Molcho L, Intrator N, Kakiashvilli E, et al. Continuous Monitoring of Mental Load During Virtual Simulator Training for Laparoscopic Surgery Reflects Laparoscopic Dexterity: A Comparative Study Using a Novel Wireless Device. Front Neurosci. 2021;15: 694010. 35126032 10.3389/fnins.2021.694010 PMC8811150

[58] Simmons C, Marsden M, Sadek S, Greenhalgh R, Lendrum R, Perkins Z. 2995 Assessment of cognitive load in clinicians performing pre-hospital REBOA: development and validation of a novel prehospital assessment tool. Emerg Med J. 2024Oct 1;41(Suppl 1):A8.

[59] Clarke S, Horeczko T, Cotton D, Bair A. Heart rate, anxiety and performance of residents during a simulated critical clinical encounter: a pilot study. BMC Med Educ. 2014Jul;27(14):153. 10.1186/1472-6920-14-153 PMC4131479 25064689

[60] Solhjoo S, Haigney MC, McBee E, van Merrienboer JJG, Schuwirth L, Artino AR, et al. Heart Rate and Heart Rate Variability Correlate with Clinical Reasoning Performance and Self-Reported Measures of Cognitive Load. Sci Rep. 2019Oct 11;9(1):14668. 31604964 10.1038/s41598-019-50280-3 PMC6789096

[61] Morton S, Spurgeon Z, Sherren P, Durge N. Pushing Yourself to the Maximum: What Do Prehospital Interventions Do to the Heart Rates of the Prehospital Team Involved? A Case Report. Air Med J [Internet]. 2023 Feb 16 [cited 2023 Apr 9]; Available from: https://www.sciencedirect.com/science/article/pii/S1067991X23000317 10.1016/j.amj.2023.01.008 37150576

[62] Prichard RS, O’Neill CJ, Oucharek JJ, Holmes CYV, Delbridge LW, Sywak MS. A prospective study of heart rate variability in endocrine surgery: surgical training increases consultant’s mental strain. J Surg Educ. 2012;69(4):453–8. 22677581 10.1016/j.jsurg.2012.04.002

[63] Sexton K, Johnson A, Gotsch A, Hussein AA, Cavuoto L, Guru KA. Anticipation, teamwork and cognitive load: chasing efficiency during robot-assisted surgery. BMJ Qual Saf. 2018Feb;27(2):148–54. 28689193 10.1136/bmjqs-2017-006701 PMC5952358

[64] P. Sarkar, K. Ross, A. J. Ruberto, D. Rodenburg, P. Hungler, A. Etemad. Classification of Cognitive Load and Expertise for Adaptive Simulation using Deep Multitask Learning. 2019 8th International Conference on Affective Computing and Intelligent Interaction (ACII), Cambridge, UK, 2019, pp.1–7. 10.1109/ACII.2019.8925507.

[65] Inama M, Spolverato G, Impellizzeri H, Bacchion M, Creciun M, Casaril A, et al. Cognitive load in 3d and 2d minimally invasive colorectal surgery. Surg Endosc. 2020Jul;34(7):3262–9. 32239306 10.1007/s00464-020-07524-5

[66] Dias RD, Conboy HM, Gabany JM, Clarke LA, Osterweil LJ, Avrunin GS, et al. Development of an Interactive Dashboard to Analyze Cognitive Workload of Surgical Teams During Complex Procedural Care. IEEE Int Inter-Discip Conf Cogn Methods Situat Aware Decis Support IEEE Int Multi-Discip Conf Cogn Methods Situat Aware Decis Support. 2018Jun;2018:77–82. 10.1109/COGSIMA.2018.8423995 PMC6289194 30547096

[67] Bertolaccini L, Viti A, Terzi A. Ergon-trial: ergonomic evaluation of single-port access versus three-port access video-assisted thoracic surgery. Surg Endosc. 2015Oct;29(10):2934–40. 25515979 10.1007/s00464-014-4024-6

[68] AlJamal YN, Baloul MS, Mathis KL, Dozois EJ, Kelley SR. Evaluating Non-operative Robotic Skills in Colorectal Surgical Training. J Surg Res. 2021Apr;260:391–8. 33261853 10.1016/j.jss.2020.11.007

[69] Mah E, Yu J, Deck M, Lyster K, Kawchuk J, Turnquist A, et al. Immersive Video Modeling Versus Traditional Video Modeling for Teaching Central Venous Catheter Insertion to Medical Residents. Cureus. 2021Mar 2;13(3): e13661. 33824812 10.7759/cureus.13661 PMC8017344

[70] Wadhera R, Henrickson S, Burkhart H, Greason K, Neal J, Levenick K, et al. Is the "sterile cockpit’’ concept applicable to cardiovascular surgery critical intervals or critical events? The impact of protocol-driven communication during cardiopulmonary bypass. J Thorac Cardiovasc Surg. 2010Feb;139(2):312–9. 20106395 10.1016/j.jtcvs.2009.10.048

[71] Horner RD, Szaflarski JP, Ying J, Meganathan K, Matthews G, Schroer B, et al. Physician work intensity among medical specialties: emerging evidence on its magnitude and composition. Med Care. 2011Nov;49(11):1007–11. 21897300 10.1097/MLR.0b013e31822dcdc7

[72] Dias RD, Zenati MA, Stevens R, Gabany JM, Yule SJ. Physiological synchronization and entropy as measures of team cognitive load. J Biomed Inform. 2019Aug;96: 103250. 31295623 10.1016/j.jbi.2019.103250 PMC7226673

[73] Talamini S, Halgrimson WR, Dobbs RW, Morana C, Crivellaro S. Single port robotic radical prostatectomy versus multi-port robotic radical prostatectomy: A human factor analysis during the initial learning curve. Int J Med Robot Comput Assist Surg MRCAS. 2021Apr;17(2): e2209. 10.1002/rcs.2209 33320437

[74] Kennedy-Metz LR, Wolfe HL, Dias RD, Yule SJ, Zenati MA. Surgery Task Load Index in Cardiac Surgery: Measuring Cognitive Load Among Teams. Surg Innov. 2020Dec;27(6):602–7. 32938323 10.1177/1553350620934931 PMC7744397

[75] Ruberto AJ, Rodenburg D, Ross K, Sarkar P, Hungler PC, Etemad A, et al. The future of simulation-based medical education: Adaptive simulation utilizing a deep multitask neural network. AEM Educ Train. 2021Jul;5(3): e10605. 34222746 10.1002/aet2.10605 PMC8155693

[76] Weigl M, Antoniadis S, Chiapponi C, Bruns C, Sevdalis N. The impact of intra-operative interruptions on surgeons’ perceived workload: an observational study in elective general and orthopedic surgery. Surg Endosc. 2015Jan;29(1):145–53. 24986016 10.1007/s00464-014-3668-6

[77] Schuetz M, Gockel I, Beardi J, Hakman P, Dunschede F, Moenk S, et al. Three different types of surgeon-specific stress reactions identified by laparoscopic simulation in a virtual scenario. Surg Endosc. 2008May;22(5):1263–7. 17943357 10.1007/s00464-007-9605-1

[78] Avrunin GS, Clarke LA, Conboy HM, Osterweil LJ, Dias RD, Yule SJ, et al. Toward Improving Surgical Outcomes by Incorporating Cognitive Load Measurement into Process-Driven Guidance. Softw Eng Healthc Syst SEHS IEEEACM Int Workshop On. 2018May;2018:2–9. 10.1145/3194696.3194705 PMC6103223 30140792

