# Supplementary material for: A scoping review of cognitive load assessment tools suitable for clinicians performing REBOA
Source: Scand J Trauma Resusc Emerg Med. 2025 Jul 9;33:121. doi: 10.1186/s13049-025-01408-0 (PMC12239420; doi:10.1186/s13049-025-01408-0)
Supplement: Supplementary file 1 — Supplementary Material 1 [file 13049_2025_1408_MOESM1_ESM.docx]

**Supplementary File 1: Search Strategy for Systematic Review**

1. **Overview:** This supplementary document details the search strategy used to identify studies for inclusion in this systematic review. It includes database-specific strategies, keywords, and search filters applied.
2. **Databases:** PubMed, Embase, Scopus, Web of Science, Cochrane Library, IEEExplore
3. **Additional Sources:** Grey literature, Systematic reviews, Reference searching
4. **Search Date:** January 2023
5. **Key Words Search:** (methods OR ways OR approach OR techniques OR tools OR modes) AND (assessing OR measuring OR monitoring OR gauging OR “subjective measurement” OR “objective measurement” OR “NASA task load index” OR “NASA-TLX” OR “Surgery task load index” OR “SURG-TLX” OR “subjective mental effort questionnaire” OR “SMEQ” OR “multiple resource questionnaire” OR “MRQ” OR “subjective workload assessment” OR “SWAT” OR “heart rate variability” OR “eye-tracking” OR “eye tracking” OR “blink rate” OR “blink-rate” OR gaze OR “eye-fixation” OR “eye fixation” OR “pupil dilation” OR electroencephalography OR “EEG” OR “functional near-infrared spectroscopy” OR “skin conductance response” OR electromyography OR “EMG” OR “heat flux” OR “facial temperature” OR “reaction time” OR “visual detection rate” OR “task precision” OR “written task” OR “wearable devices”) AND (physicians OR clinicians OR team OR “team leaders” OR surgeons OR specialists) AND (“cognitive load” OR “cognitive workload” OR “cognitive bandwidth” OR “cognitive load theory” OR “mental strain” OR “mental effort”) AND (“clinical medicine” OR “emergency medicine” OR “accident & emergency medicine” OR surgery OR “prehospital medicine” OR “pre-hospital medicine” OR “trauma medicine” OR “trauma surgery” OR “haemorrhage control” OR “hemorrhage control”)
6. **Filters Applied:** Language: English
7. **Adaptation Across Databases:** Search strategy was adapted for each database, considering variations in field tags and syntax.
8. **Grey Literature and Additional Sources:** References from systematic reviews captured in the general search were reviewed for additional sources. Other sources were obtained in an opportunistic manner and included if met the study criteria.
9. **PRISMA:** PRISMA Guidelines were adhered to throughout.
