# Supplementary material for: A scoping review of cognitive load assessment tools suitable for clinicians performing REBOA
Source: Scand J Trauma Resusc Emerg Med. 2025 Jul 9;33:121. doi: 10.1186/s13049-025-01408-0 (PMC12239420; doi:10.1186/s13049-025-01408-0)
Supplement: Supplementary file 2 — Supplementary Material 2: [62–78] [file 13049_2025_1408_MOESM2_ESM.docx]

**Additional File 2: Summary of Included Literature and Risk of Bias Assessment**

| **Author/Date** | **Title** | **Country** | **Simulation or Real-Life** | **Analogous Clinical Environment** | **Procedure** | **Participants** | **Cognitive Load Measurement Tools** | | **MERSQ** |
| --- | --- | --- | --- | --- | --- | --- | --- | --- | --- |
|  |  |  |  |  |  |  | **Subjective/Self-report** | **Objective/Real-time** |  |
| Böhm et al. 2001(32) | “A prospective randomized trail on heart rate variability of the surgical team during laparoscopic and conventional sigmoid resection” | Germany | Real-life | Theatre | Laparoscopic sigmoid resection | 4 surgeons |  | HRV | 15 |
| Prichard et al. 2012 (62) | “A prospective study of heart rate variability in endocrine surgery: surgical training increases consultant’s mental strain” | Australia | Real-life | Theatre | Thyroid lobectomy | 5 surgeons |  | HRV | 14.5 |
| Pimentel et al. 2019 (14) | “A wearable approach for intraoperative physiological stress monitoring of multiple cooperative surgeons” | USA | Real-life | Theatre | Cerebral aneurysm clipping | 2 surgeons | Likert scale, SURG-TLX | HRV | 13.5 |
| Yang et al. 2022 (34) | “An adaptive human-robotic interaction architecture for augmenting surgery performance using real-time workload sensing-demonstration of semi-autonomous suction tool” | USA | Simulation | Theatre | Haemorrhage control | 10 surgeons, 10 students |  | Secondary task analysis (reaction time), EEG, eye-tracking | 13 |
| López-Cano et al. 2012 (26) | “An ergonomic study of single-port versus multi-port laparoscopic mesh insertion for ventral hernia repair” | Spain | Simulation | Theatre | Mesh hernia repair | 10 surgeons | SMEQ |  | 12 |
| Sexton et al. 2018 (63) | “Anticipation, teamwork and cognitive load: chasing efficacy during robot-assisted surgery” | USA | Real-life | Theatre | Robot assisted prostatectomies | 6 surgeons, 18 nurses | NASA-TLX |  | 12.5 |
| Chowriappa et al. 2015 (42) | “Augmented-reality-based skills training for robot-assisted urethrovesical anastomosis: a multi-institutional randomised controlled trial” | USA | Simulation | Theatre | Robot assisted urethrovesical anastomosis | 52 surgeons | NASA-TLX |  | 14.5 |
| Dixon et al. 2014 (43) | “Augmented real-time navigation with critical structure proximity alerts for endoscopic skull base surgery” | USA | Simulation | Theatre | Endoscopic skull base surgery | 7 surgeons | NASA-TLX |  | 14.5 |
| Shafiei et al. 2018 (33) | “Can eye tracking help explain an expert surgeon’s brain performance during robot-assisted surgery” | USA | Real-life | Theatre | Robot assisted radical cystectomy | 1 surgeon |  | Eye-tracking | 12.5 |
| P. Sarkar et al. 2019 (64) | “Classification of cognitive load and expertise for adaptive simulation using deep multitask learning” | Canada | Simulation | Emergency | Management of blunt and penetrating trauma scenarios | 5 EM physicians, 4 medical students |  | HRV, EMG, GSR | 13 |
| Frederiksen et al. 2020 (37) | “Cognitive load and performance in immersive virtual reality versus conventional virtual reality simulation training of laparoscopic surgery: a randomized trial” | Denmark | Simulation | Theatre | Laparoscopic salpingectomy | 31 surgeons |  | Secondary task analysis (reaction time) | 13.5 |
| Inama et al. 2020 (65) | “Cognitive load in 3D and 2D minimally invasive colorectal surgery” | Italy | Real-life | Theatre | Colorectal resections | 4 surgeons | NASA-TLX |  | 13.5 |
| Kapp et al. 2021 (15) | “Cognitive load in electromagnetic navigational and robotic bronchoscopy for pulmonary nodules” | USA | Simulation | Theatre | Bronchoscopy | 6 surgeons | SURG-TLX, Likert scale | Eye-tracking | 12 |
| Anderson et al. 2016 (38) | “Cognitive load in mastoidectomy skills training: virtual reality simulation and transitional dissection compared” | Denmark | Simulation | Theatre | Mastoidectomy | 40 surgeons |  | Secondary task analysis (reaction time) | 12.5 |
| Kelkar et al. 2022 (17) | “Cognitive workload, complications and visual outcomes of phacoemulsification cataract surgery: three-dimensional versus conventional microscope” | India | Real-life | Theatre | Cataract surgery | 3 surgeons | SURG-TLX | HRV, SpO2% | 14 |
| Anschuetz et al. 2019 (16) | “Comparison of 3- vs 2-Dimensional endoscopy using eye-tracking and assessment of cognitive load among surgeons performing endoscopic ear surgery” | Switzerland | Simulation | Theatre | Tympanoplasty, stapedotomy | 16 surgeons | NASA-TLX | Eye-tracking | 12 |
| Boet et al. 2017 (39) | “Debriefing decreases mental workload in surgical crisis: A randomized controlled trial” | Canada | Simulation | Emergency | Management of postoperative shock | 20 surgeons |  | Secondary task analysis (reaction time) | 12.5 |
| Dias et al. 2018 (66) | “Development of an interactive dashboard to analyse cognitive workload of surgical teams during complex procedural care” | USA | Real-life | Theatre | CABG, AVR | 2 surgeons, 2 anaesthetists, 2 perfusionists |  | HRV | 13.5 |
| Gutierrez et al. 2021 (44) | “Do preparatory online modules optimize cognitive load during simulated resuscitation scenarios?” | Canada | Simulation | Emergency | Resuscitation scenarios | 53 surgeons | Leppink questionnaire |  | 14.5 |
| Bertolaccini et al. 2015 (67) | “Ergon-trial: ergonomic evaluation of single-port access versus three-port access video-assisted thoracic surgery” | Italy | Real-life | Theatre | Video assisted thoracic surgery | 3 surgeons | NASA-TLX |  | 12.5 |
| Pappada et al. 2016 (18) | “Establishing an instrumental training environment for simulation-based training of health care providers: An initial proof of concept” | USA | Simulation | Theatre | Damage control | 1 surgeon, 1 nurse, 1 anaesthetist | NASA-TLX | EEG, HRV | 12 |
| A.I. Jamal et al. 2021 (68) | “Evaluating non-operative robotic skills in colorectal surgical training” | USA | Simulation | Theatre | Haemostasis, management of CO2 embolus | 6 surgeons | NASA-TLX |  | 12 |
| Stelter et al. 2011 (40) | “Evaluation of an image-guided navigation system in the training of functional endoscopic sinus surgeons. A prospective, randomised clinical study” | Germany | Real-life | Theatre | Endoscopic sinus surgery | 8 surgeons | HFEQ-CASS |  | 15 |
| Merkle et al. 2019 (19) | “Evaluation of attention, perception, and stress levels of clinical cardiovascular perfusionists during cardiac operations: a pilot study” | Germany | Real-life | Theatre | Cardiopulmonary bypass | 9 perfusionists | NASA-TLX | Eye-tracking | 13.5 |
| Lowe et al. 2016 (35) | “Feasibility of EEG to monitor cognitive performance during venous cannulation: EEG Distracted Intravenous Access (E-DIVA)” | UK | Simulation | Emergency | IV cannulation | 10 EM physicians, 10 medical students |  | Secondary task analysis (reaction time), EEG | 13 |
| Pluyter et al. 2014 (45) | “Immersive training: breaking the bubble and measuring the heat” | Netherlands | Simulation | Theatre | Laparoscopic cholecystectomy | 21 surgeons |  | Skin temperature | 13.5 |
| Mah et al. 2021 (69) | “Immersive video modelling versus traditional video modeling for teaching central venous catheter insertion to medical residents” | Canada | Simulation | Emergency | Central venous catheter insertion | 32 participants (surgeons, anaesthetists, EM physicians) | NASA-TLX |  | 11.5 |
| Dias et al. 2018 (20) | “Intelligent interruption management system to enhance safety and performance in complex surgical and robotic procedures” | USA | Real-life | Theatre | CABG, AVR | 1 surgeon, 1 anaesthetist, 1 perfusionist | SURG-TLX | HRV | 13.5 |
| Britt et al. 2015 (21) | “Intracoporeal suturing: transfer from fundamentals of laparoscopic surgery to cadavers results in substantial increase in mental workload” | USA | Simulation | Theatre | Intracorporeal procedures | 14 surgeons | NASA-TLX | Secondary task analysis (reaction time) | 11 |
| Vera et al. 2019 (22) | “Intraocular pressure increases after complex simulated surgical procedures in residents: an experimental study” | Spain | Simulation | Theatre | Bronchoscopy | 17 surgeons | NASA-TLX | IOP | 12.5 |
| Wadhera et al. 2010 (70) | “Is the ‘sterile cockpit’ concept applicable to cardiovascular surgery critical intervals or critical events? The impact of protocol-driven communication during cardiopulmonary bypass” | Canada | Real-life | Theatre | Cardiac surgery, cardiopulmonary bypass | 30 participants (perfusionists, nurses, surgeons) | NASA-TLX |  | 12.5 |
| Kumar et al. 2022 (27) | “Lithotomy versus prone position for perianal surgery: a randomized controlled trial” | India | Real-life | Theatre | Perianal surgery | 12 surgeons, 7 nurses | SMEQ |  | 14 |
| Bingener et al. 2014 (25) | “Modified NASA workload tool identifies physical and cognitive surgeon workload for laparoscopic procedures” | USA | Real-life | Theatre | Laparoscopic cholecystectomy | 1 surgeon | SURG-TLX |  | 15 |
| Park et al. 2022 (31) | “Objective measurement of learners’ cognitive load during simulation - based trauma team training: a pilot study” | USA | Simulation | Emergency | Trauma scenarios | 11 EM physicians/nurses |  | HRV | 12 |
| Horner et al. 2011 (71) | “Physician work intensity among medical specialities: emerging evidence on its magnitude and composition” | USA | Real-life | Emergency | Post-op care | 111 physicians | NASA-TLX, SWAT, MRQ |  | 11 |
| Dias et al. 2019 (72) | “Physiological synchronisation and entropy as measures of team cognitive load” | USA | Real-life | Theatre | CABG | 3 surgeons, 1 anaesthetist, 1 perfusionist, 1 nurse |  | HRV | 13.5 |
| Huckaby et al. 2022 (24) | “Postprocedural cognitive load measurement with immediate feedback to guide curriculum development” | USA | Real-life | Theatre | Bronchoscopy, endoscopy, thoracoscopy | 11 surgeons | NASA-TLX |  | 12.5 |
| Fenik et al. 2013 (46) | “Prepackaged central line kits reduce procedural mistakes during central line insertion: a randomized controlled prospective trial” | Germany | Simulation | Emergency | Central venous catheter insertion | 30 EM physicians/medical students |  | Procedure error rate | 14 |
| Weinger et al. 2000 (28) | “Quantitative description of the workload associated with airway management procedures” | USA | Real-life | Theatre | Airway procedures | 241 anaesthetists | Likert scale |  | 11.5 |
| Heemskerk et al. 2014 (41) | “Relax, it’s just laparoscopy! A prospective randomized trial on heart rate variability of the surgeon in robot-assisted versus conventional laparoscopic cholecystectomy” | Netherlands | Real-life | Theatre | Laparoscopic cholecystectomy | 1 surgeon |  | HRV | 15 |
| Grochola et al. 2018 (29) | “Robot-assisted single-site compared with laparoscopic single-incision chelecystectomy for benign gallbladder disease: results of a single-blinded randomized controlled trial” | Switzerland | Real-life | Theatre | Laparoscopic cholecystectomy | 1 surgeon | SMEQ |  | 14 |
| Kennedy-Metz et al. 2020 (36) | “Sensors for continuous monitoring of surgeon’s cognitive workload in the cardiac operating room” | USA | Real-life | Theatre | AVR | 1 surgeon |  | fNIR, HRV | 13.5 |
| Talamini et al. 2021 (73) | “Single port robotic radical prostatectomy versus multi-port robotic radical prostatectomy: a human factor analysis during the initial learning curve” | Italy & USA | Real-life | Theatre | Radical prostatectomy | 1 surgeon | NASA-TLX, SURG-TLX, OTAS |  | 13.5 |
| Joseph et al. 2016 (23) | “Stress among surgical attending physicians and trainees: a quantitative assessment during trauma activation and emergency surgeries” | USA | Real-life | Theatre | Damage control | 22 surgeons | STAI, NASA-TLX | HRV | 12.5 |
| Kennedy-Metz et al. 2020 (74) | “Surgery task load index in cardiac surgery: measuring cognitive load among teams” | USA | Real-life | Theatre | Cardiopulmonary bypass | 3 surgeons, 5 anaesthetists, 3 perfusionists | SURG-TLX |  | 13.5 |
| Ruberto et al. 2021 (75) | “The future of simulation-based medical education: adaptive simulation utilizing a deep multitask neural network” | Canada | Simulation | Emergency | Asthma exacerbation management | 2 EM physicians, 2 medical students |  | HRV, GSR | 11 |
| Weigl et al. 2015 (76) | “The impact of intra-operative interruptions on surgeons’ perceived workload: an observational study in elective general and orthopedic surgery” | Germany | Real-life | Theatre | General and orthopaedic surgery | 63 surgeons | NASA-TLX |  | 14 |
| Schuetz et al. 2008 (77) | “Three different types of surgeon-specific stress reactions identified by laparoscopic simulation in a virtual scenario” | Germany | Simulation | Theatre | Laparoscopic cholecystectomy | 18 surgeons |  | GSR | 11.5 |
| Avrunin et al. 2018 (78) | “Toward improving surgical outcomes by incorporating cognitive load measurement into process-driven guidance” | USA | Real-life | Theatre | CABG, AVR | 1 surgeon, 1 anaesthetist, 1 perfusionist |  | HRV | 9 |
